# MicroRNA-21 regulates Osteogenic Differentiation of Periodontal Ligament Stem Cells by targeting Smad5

**DOI:** 10.1038/s41598-017-16720-8

**Published:** 2017-11-30

**Authors:** Fulan Wei, Shuangyan Yang, Qingyuan Guo, Xin Zhang, Dapeng Ren, Tao Lv, Xin Xu

**Affiliations:** 1 Department of Orthodontics, School of Stomatology, Shandong University, Jinan, People’s Republic of China; 2 Shandong Provincial Key Laboratory of oral tissue regeneration, School of Stomatology, Shandong University, Jinan, People’s Republic of China; 3 Department of Implantology, School of Stomatology, Shandong University, Jinan, People’s Republic of China

## Abstract

Human periodontal ligament stem cells (hPDLSCs) are mesenchymal stem cells (MSCs) derived from dental and craniofacial tissues that exhibit high potential for differentiation into osteoblasts. Recently, microRNAs (miRNAs) have been established to play important roles in MSC osteogenesis. In the current study, we report that miR-21 was down-regulated in osteogenically differentiated PDLSCs. Overexpression of miR-21 significantly inhibited osteogenesis of hPDLSC, whereas its inhibition demonstrated the opposite effects. Furthermore, SMAD family member 5 (Smad5) was predicted to be a downstream target of miR-21 and was shown to undergo up-regulation in PDLSCs induced toward osteogenesis. Moreover, Smad5 and Runx2, which are the critical transcription factors in osteogenic differentiation, were predicted to be targets of miR-21. Suppression of miR-21 expression increased the level of Smad5 *in vitro* and during *in vivo* transplantation experiments. Furthermore, suppression of Smad5 inhibited osteogenic differentiation and decreased the protein level of Runx2. Taken together, these results suggested that miR-21 be mechanistically implicated in the regulation of osteogenic differentiation of hPDLSCs by targeting Smad5.

## Introduction

Mesenchymal stem cells (MSCs) play a critical role in tissue regeneration due to their ability to self-renew and differentiate into multiple cell lineages. MSCs have been employed in clinical trials as a potential therapeutic solution to severe refractory diseases. Recently, new populations of MSCs from dental and craniofacial tissues, including periodontal ligament stem cells (PDLSCs) and other dental stem cells, have been proposed as suitable cell sources for tooth and periodontal tissue regeneration^1–3^. Our previous studies have demonstrated the feasibility of regenerating periodontal tissues and bioengineered tooth root (bio-root) structure in miniature pigs^4–6^. Despite the intense interest in PDLSC-based therapies, the clinical potential of these cells has been severely limited by a lack of understanding of the molecular mechanism that underlies their directed differentiation. MSC differentiation involves a series of complex pathways that are regulated at both the transcriptional and post-transcriptional levels. Previous studies have found runt-related transcription factor 2 (Runx2) and osterix (OSX) to be important transcriptional regulators of osteoblast differentiation^7,8^. Recently, it was revealed that small, noncoding microRNAs (miRNAs) could modulate the differentiation potential of stem cells by post-transcriptionally targeting factors implicated in stem cell maintenance^9^. Increasing evidence also suggested that miRNAs could also contribute to the modulation of osteogenic differentiation of various cell types and bone formation^10,11^.

MicroRNA-21 (miR-21) has been found to be overexpressed in most epithelial cancer tissues and therefore believed to play a pivotal role in the progression of a wide variety of malignancies^12^. Furthermore, inhibition of miR-21 was found to suppress proliferation and promote apoptosis of cancer cells^13^. Additional research demonstrated that miR-21 could down-regulate the expression of putative tumor-suppressive genes, such as programmed cell death 4 (PDCD4)^14^, phosphatase and tensin (PTEN)^15^, maspin^16^, NFIB^17^, etc. Our previous study revealed that miR-21 enhanced stretch-induced osteogenic differentiation in human periodontal ligament stem cells (hPDLSCs)^18^. In this study, we sought to examine the mechanistic role of miR-21 in the osteogenic differentiation of hPDLSCs and identify its regulatory targets. Our data indicated that miR-21, which is down-regulated during osteogenic differentiation of hPDLSCs, inhibited the osteogenic differentiation process of hPDLSCs by targeting SMAD family member 5 (Smad5). This information is expected to provide novel insights into differentiation of MSCs in tissue regeneration applications.

## Results

### MiR-21 decreased during osteogenic differentiation of hPDLSCs

**Figure 1:**
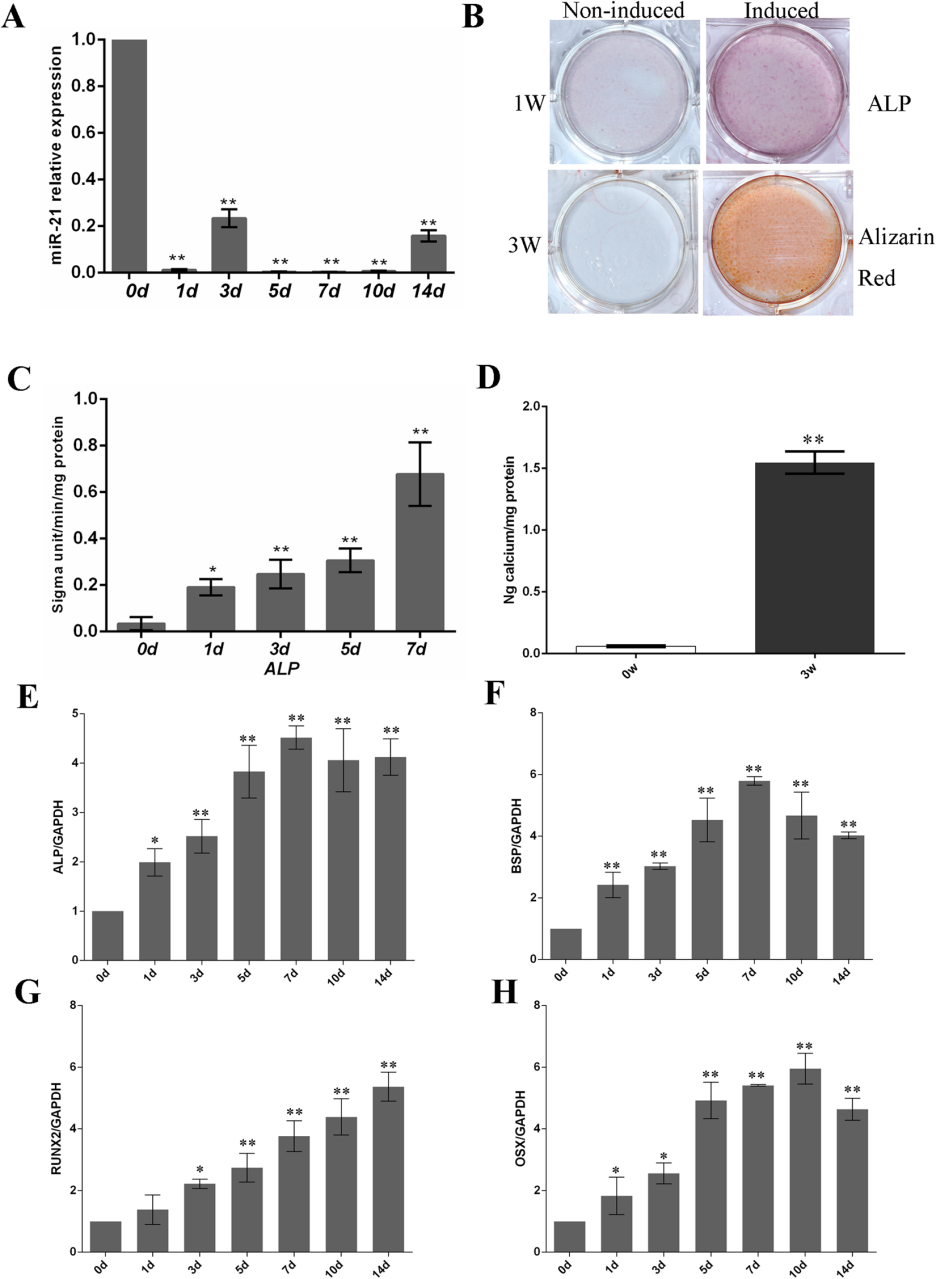
MiR-21 was down-regulated during the osteogenic differentiation of hPDLSCs. Osteogenic differentiation of hPDLSCs was induced for 14 days. miRNAs were prepared at day 0, 1, 3, 5, 7, 10 and 14 after the induction and used immediately for real-time PCR analysis. The relative quantities of the PCR-amplified products were determined by an image analyzer. Each data point was shown as the mean ± SD of the ratio of the miR-21 level to that on day 0 (n = 4). *p < 0.05, **P < 0.01 compared with the corresponding control sample prepared at the same time point. (**A**) miR-21 level was suppressed in osteoblastically differentiated hPDLSCs. (**B**) ALP staining and Alizarin Red S staining were performed at day 7 and at day 21, respectively, both of which indicated the occurrence of hPDLSC osteogenesis. (**C**) Quantitation of ALP activity level over the course of differentiation. (**D**) Quantification of calcium level at week 0 and 3. (**E–H**) Osteoblast differentiation was confirmed by qRT-PCR analysis of osteoblast marker genes, including ALP (**E**), BSP (**F**), Runx2 (**G**) and OSX (**H**). GAPDH was used as an internal standard (n = 3 for all experiments).

We assessed the trend of miR-21 expression in relation to osteogenesis in hPDLSCs. Osteogenic differentiation of hPDLSCs was induced in a standard osteoblast induction medium and the osteoblastic phenotype was evidenced by increased alkaline phosphatase (ALP) activity (Fig. 1B,C) and matrix mineralization as confirmed via Alizarin Red S staining (Fig. 1B,D). Subsequently, the levels of miR-21 (Fig. 1A) and various osteogenesis-related genes, such as ALP, bone Sialoprotein (BSP), Runx2 and OSX (Fig. 1E–H), were measured by qRT-PCR at days 1, 3, 5, 7, 10, 14 following the induction. On the one hand, the results provided further proof for the occurrence of osteogenic differentiation by indicating that all four osteogenic genes were up-regulated. These findings were in accordance with the previous reports on hPDLSC differentiation into osteoblasts^19,20^. On the other hand, miR-21 was found to be down-regulated in a time-dependent manner after the induction of osteogenic differentiation in hPDLSCs (Fig. 1A).

### Overexpression of miR-21 decreased the osteogenic differentiation of hPDLSCs

**Figure 2:**
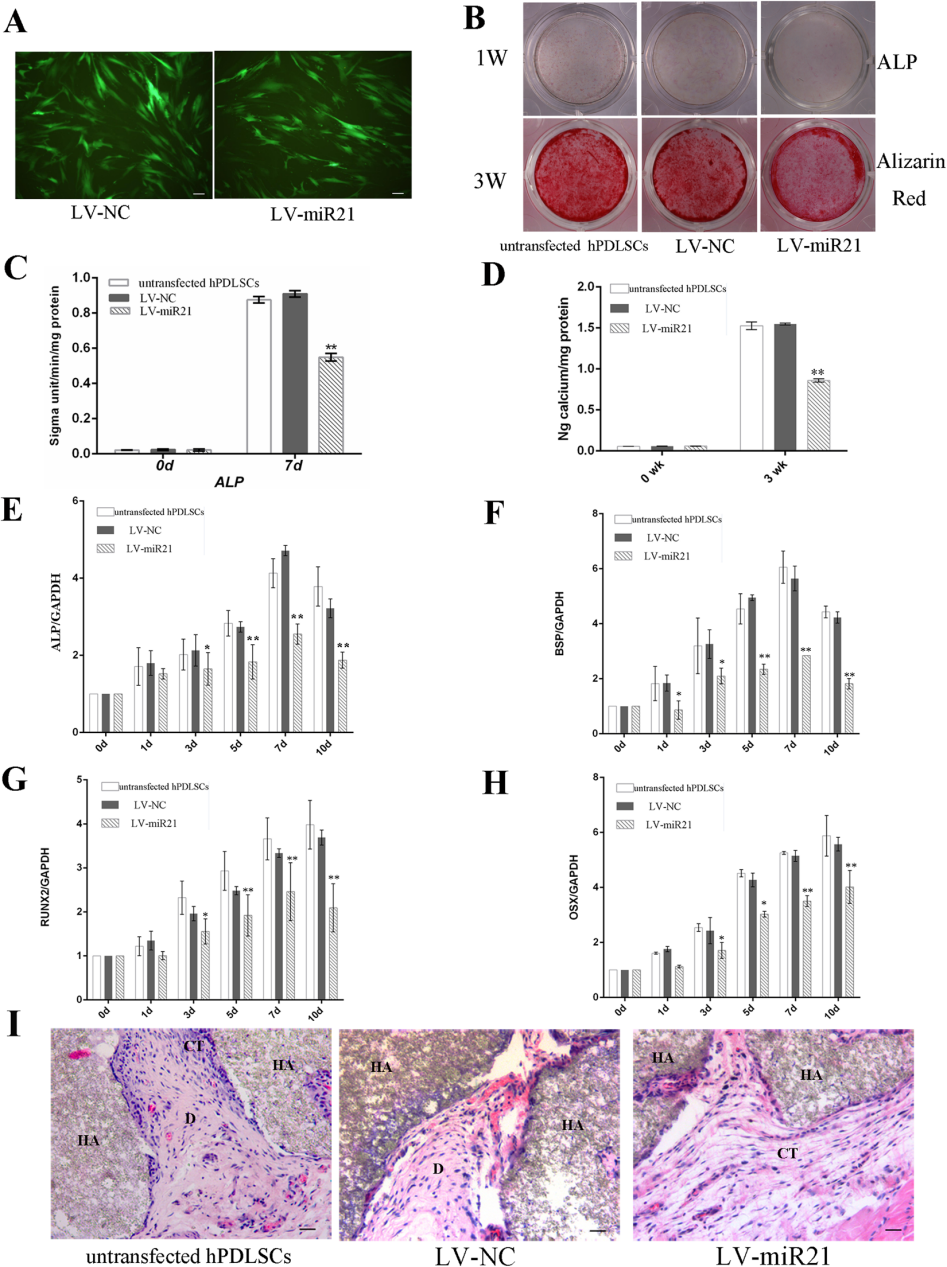
Overexpression of miR-21 inhibited the osteogenic differentiation of hPDLSCs. (**A**) Fluorescence microscopic analysis of hPDLSCs transfected with the empty vector (left, the LV-NC group) or pGC-LV-pre-miR-21-GFP (right, the overexpression group), showing no obvious difference in their morphology. (magnification, × 200). (**B**) ALP staining and Alizarin Red S staining of the three experiment groups. (**C**, **D**) Quantitation of ALP (**C**) and calcium (**D**) levels. (**E**-**H**) Real-time RT-PCR analysis of the expression levels of ALP (**E**), BSP (**F**), Runx2 (**G**) and OSX (**H**) in the three experiment groups. *p < 0.05, **P < 0.01 compared with untransfected hPDLSCs.(**I**) H&E staining provided evidence of mineralization eight weeks after the nude mice were transplanted with hPDLSCs overexpressing miR-21. D, bone/dentin-like matrix tissue; HA, hydroxyapatite tricalcium carrier; CT, connective tissues. Scale bar indicated a distance of 100 μm.

To assess the role of miR-21 in the osteogenic differentiation of hPDLSCs, we transfected hPDLSCs with a lentiviral construct pGC-LV-pre-miR-21-GFP (referred to as the overexpression group hereafter) designed to stimulate the production of miR-21. A similar construct excluding the miR-21 sequence, pGC-LV-miR-NC-GFP, was used as a control (referred to as the LV-NC group hereafter). After lentiviral transduction, over 90% of the hPDLSCs in the overexpression group expressed green fluorescent protein (GFP) and exhibited a morphology similar to that displayed in the LV-NC group (Fig. 2A).

To elucidate the effect of miR-21 overexpression on the efficiency of hPDLSC differentiation, the transfected hPDLSCs were induced to differentiate along osteogenic lineages, followed by measuring the activity of ALP, an early marker for osteo/dentinogenic differentiation, one week after the induction. It was shown that ALP activity level was much lower in the hPDLSCs overexpressing miR-21 compared to the LV-NC and the uninfected cells (Fig. 2B,C). Three weeks after induction, Alizarin Red S staining and calcium quantitation assays were performed, which revealed a decreased extent of osteoblastic mineralization in the overexpression group in comparison to both the LV-NC group and the uninfected hPDLSCs (Fig. 2B,D). Consistent with these results, overexpression of miR-21 was also confirmed by qRT-PCR analysis to lead to attenuated induction of several osteo/dentinogenic marker genes, including ALP, BSP, Runx2 and OSX, in hPDLSCs (Fig. 2E–H).

To ascertain whether similar effects could also be found *in vivo*, cell suspension and hydroxyapatite/tricalcium phosphate (HA/TCP) ceramic particles were mixed together and implanted subcutaneously into the dorsal surface of nude mice. Hematoxylin and eosin (H&E) staining was performed to analyze morphological changes. As illustrated in Fig. 2I, the implanted hPDLSCs overexpressing miR-21 generated less bone/dentin-like mineralized tissues than the cells that were either transfected with the empty lentiviral vector or not infected.

### Inhibition of miR-21 increased osteogenic differentiation of hPDLSCs

**Figure 3:**
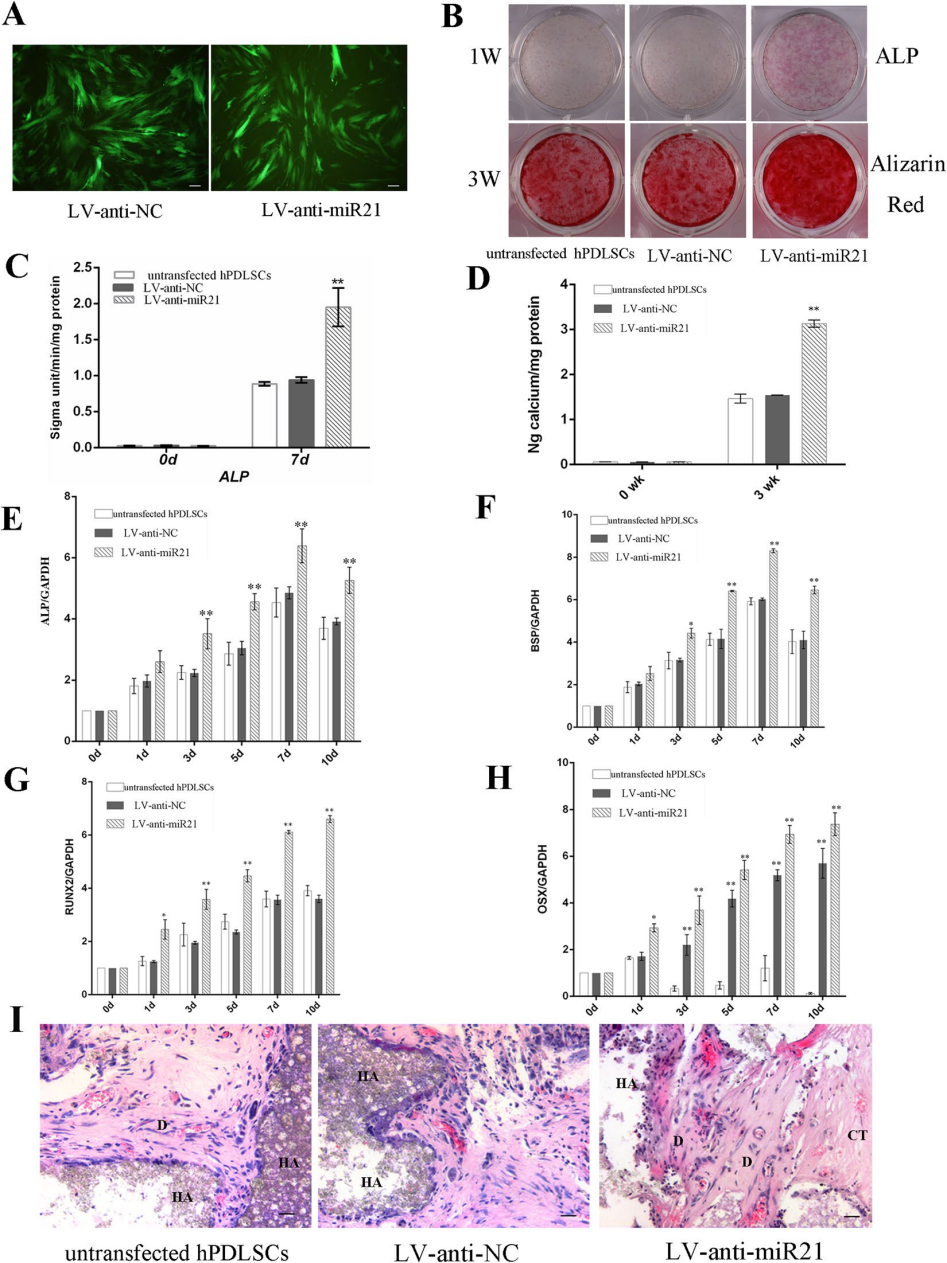
Inhibition of miR-21 stimulated the osteogenic differentiation of hPDLSCs. (**A**) Fluorescence microscopic analysis of hPDLSCs transfected with the empty vector (left, the LV-anti-NC group) or pGC-LV-anti-miR-21-GFP (right, the inhibition group), showing no obvious difference in their morphology. (magnification, × 200). (**B**) ALP staining and Alizarin Red S staining of the three experiment groups. (**C**, **D**) Quantitation of ALP (**C**) and calcium (**D**) levels. (**E**-**H**) Real-time RT-PCR analysis of the expression levels of ALP (**E**), BSP (**F**), Runx2 (**G**) and OSX (**H**) in the three experiment groups. *p < 0.05, **P < 0.01 compared with untransfected hPDLSCs. (**I**) H&E staining provided evidence of mineralization eight weeks after the nude mice were transplanted with hPDLSCs inhibiting miR-21. D, bone/dentin-like tissue; HA, hydroxyapatite tricalcium carrier; CT, connective tissues. Scale bar indicated a distance of 100 μm.

We next investigated whether suppression of miR-21 expression could lead to effects opposite to those mentioned above. For miR-21 knockdown, hPDLSCs were transfected with the lentiviral construct pGC-LV-anti-miR-21-GFP (referred to as the knockdown group hereafter). After lentiviral transduction, over 90% of the hPDLSCs in the inhibition group expressed green fluorescent protein (GFP) and exhibited a morphology similar to that displayed in the LV-anti-NC group (Fig. 3A). As expected, the down-regulation of miR-21 resulted in a series of molecular and functional changes that were antithetical to what was observed when overexpression of miR-21 was induced, including elevated ALP activity (Fig. 3B,C) and a greater extent of mineralization (Fig. 3B,D) in the hPDLSCs transfected with anti-miR-21 compared to the LV-anti-NC and untransfected cells. Consistent with these reversals, qRT-PCR analysis showed that mRNA levels of ALP, BSP, Runx2 and OSX were dramatically higher in the knockdown group than in the LV-anti-NC group or in untransfected cells after the induction (Fig. 3E–H). *In vivo* implantation experiments also demonstrated that the miR-21-knockdown hPDLSCs generated more bone/dentin-like mineralized tissues than the cells that were either transfected with pGC-LV-anti-miR-NC-GFP or not infected (Fig. 3I).

### MiR-21 inhibited osteogenic differentiation in PDLSCs through Smad5

**Figure 4:**
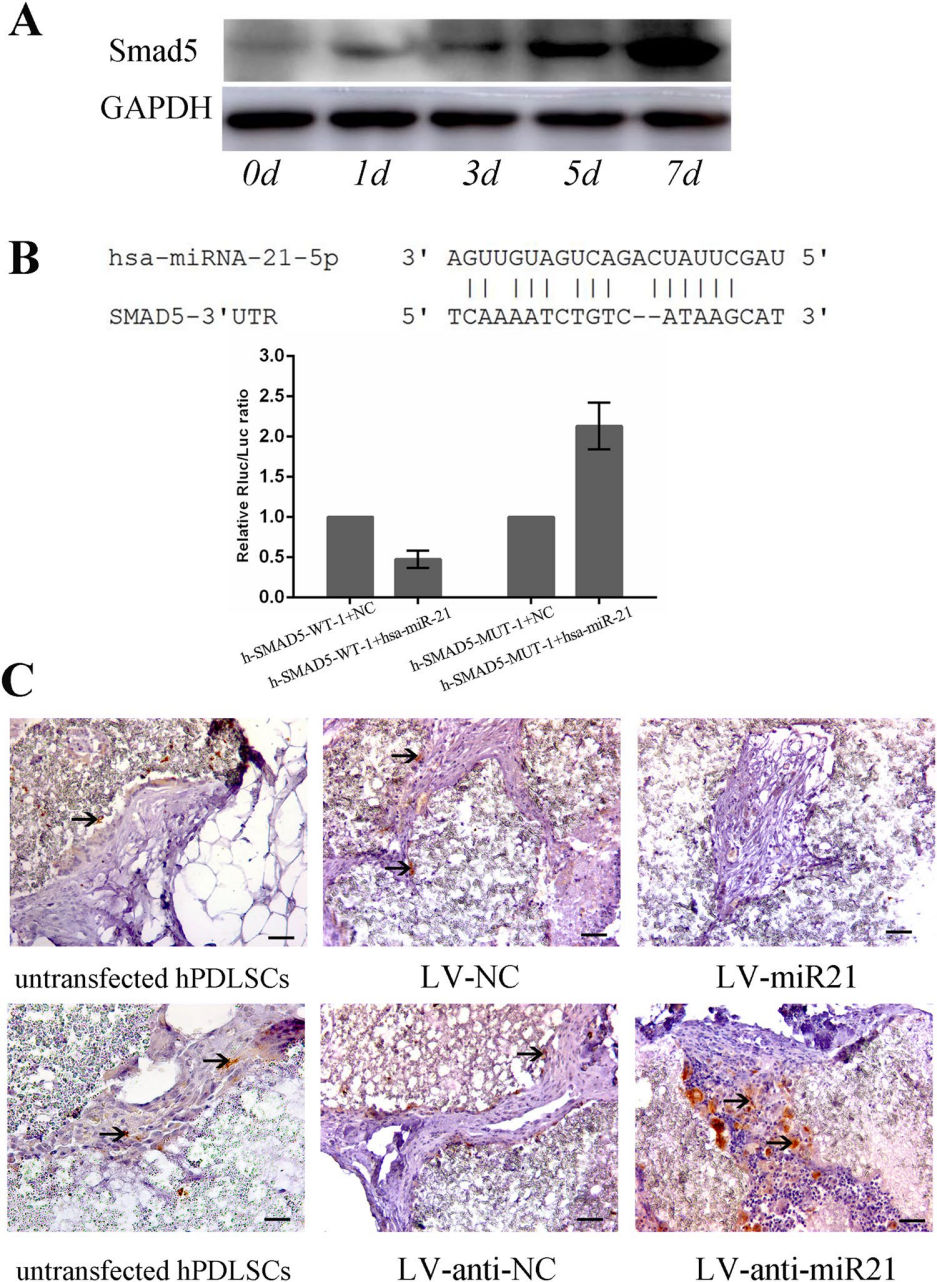
MiR-21 regulates the osteogenic differentiation of hPDLSCs through targeting Smad5. (**A**) Western blot analysis showed an up-regulation of Smad5 during the induced osteogenic differentiation of hPDLSCs. (**B**) miR-21 directly targets Smad5 mRNA. The 3′UTR element of Smad5 mRNA is partially complementary to the sequence of miR-21. The luciferase activities were measured at 48 h after transfection with pre-miR-21. The values represent the mean±SD for triplicate samples from a representative experiment. (**C**) The expression of Smad5 was visualized by immunohistochemical staining. Smad5 was strongly expressed in hPDLSCs transfected with anti-miR21 (bottom right) compared to both the control group (bottom middle) and uninfected cells (bottom left). In contrast, Smad5 was down-regulated in miR-21-overexpressed hPDLSCs (top right).

To explore the molecular mechanism through which miR-21 inhibited osteogenic differentiation in PDLSCs, we performed bioinformatics analysis on miRGen 2.0 (www.targetscan.org, www.microrna.org, http://pictar.bio.nyu.edu) to search for its potential downstream target genes. The search led to the identification of 12 putative targets of miR-21 that could potentially be related to osteoblastic differentiation (Table 1). One of the hits was Smad5, a protein with an established role in MSC osteogenesis^21^. As a result, we analyzed the protein level of Smad5 in hPDLSCs undergoing osteogenic differentiation. Western blot confirmed that Smad5 was up-regulated on a protein level during the osteoblastic differentiation of uninfected hPDLSCs (Fig. 4A). On the other hand, Luciferase assay report indicated Smad5 was a potential target of miR-21 and miR-21 directly targeted Smad5 mRNA. Hsa-miR-21mimic had a significant down-regulation effect on SMAD5 wild-type reported fluorescence, and after the mutation of its predicted target site, the reported fluorescence has been restored (Fig. 4B). The sequencing results of the Smad5-mutant vectors were showed in Fig. S1. Mutant vector of Smad5-MUT-1 has an obvious effect. Furthermore, according to western blot analysis, after 7 days of osteogenic induction, Smad5 expression was decreased in hPDLSCs overexpressing miR-21 compared to the ones that were either transfected with pGC-LV-miR-NC-GFP or not infected, but increased in the cells in which miR-21 expression was artificially suppressed (Fig. S2).

**Table 1:** The potential target that related to osteoblast differentiation of miR-21 based on the KEGG pathway analyses.

miRNA	Target name	Possible function of targets
hsa-miR-21	IL6R	positive regulation of osteoblast differentiation
hsa-miR-21	IL6ST	positive regulation of osteoblast differentiation
hsa-miR-21	Smad5	positive regulation of osteoblast differentiation
hsa-miR-21	BMP7	positive regulation of osteoblast differentiation
hsa-miR-21	BMPR1B	positive regulation of osteoblast differentiation
hsa-miR-21	BMPR2	positive regulation of osteoblast differentiation
hsa-miR-21	ACVR2B	positive regulation of osteoblast differentiation
hsa-miR-21	CDK6	negative regulation of osteoblast differentiation
hsa-miR-21	SKI	negative regulation of osteoblast differentiation
hsa-miR-21	SOX2	negative regulation of osteoblast differentiation
hsa-miR-21	APC	regulation of osteoblast differentiation
hsa-miR-21	MITF	regulation of osteoclast differentiation

In addition, immunohistochemistry analysis of the hPDLSCs transfected and implanted into the mouse model indicated a significantly higher expression level of Smad5 in the miR-21-knockdown cells compared to the LV-anti-NC and uninfected cells. On the other hand, overexpression of miR-21 resulted in markedly attenuated expression of Smad5 (Fig. 4C).

### Effects of Smad5 on the osteogenic differentiation of hPDLSCs

**Figure 5:**
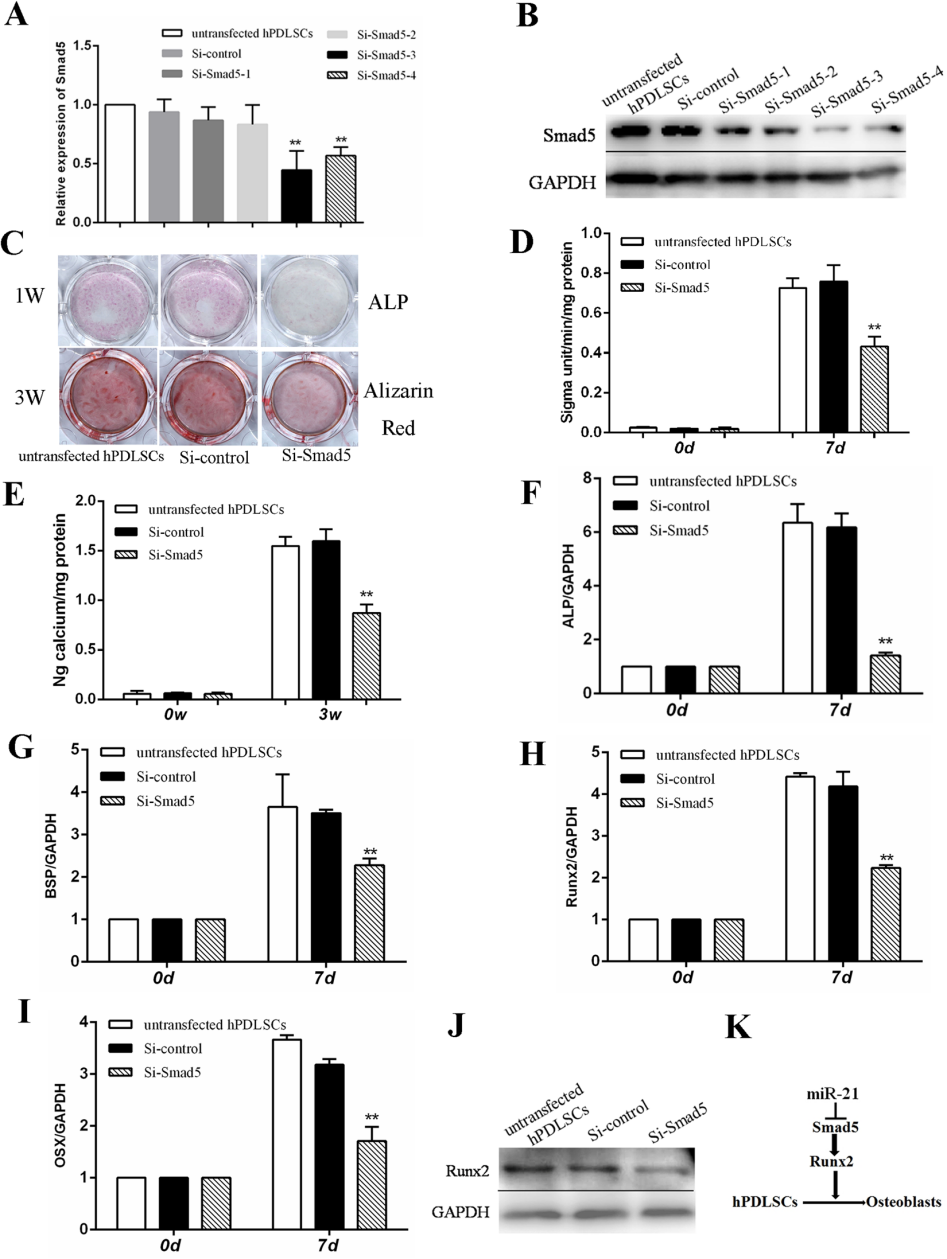
Effects of Smad5 on the osteogenic differentiation of hPDLSCs. PDLSCs were transfected with control siRNA, siRNA-Smad5 for 72 h. (**A** and **B**) Real-time RT-PCR and western blot results showed that Smad5 was silenced in Si-Smad5-3 transfected cells. (**C**) ALP staining and Alizarin Red S staining of the three experiment groups. (**D** and **E**) Quantitation of ALP (**D**) and calcium (**E**) levels. (**F**-**I**) Real-time RT-PCR analysis of the expression levels of ALP (**F**), BSP (**G**), Runx2 (**H**), OSX (**I**) in the three experiment groups. The values represent the mean ± SD for triplicate samples from a representative experiment (*p < 0.05, **P < 0.01 compared with untransfected hPDLSCs.). (**J**) Western blot analysis indicating the expression levels of Runx2 in different experiment groups. Runx2 expression was decreased in Smad5-knockdown hPDLSCs (rightmost, Si-Smad5). (**K**) A schematic illustration of the hypothetical regulatory pathway through which miR-21 modulates hPDLSC osteogenic differentiation via Smad5.

To determine the role of Smad5 on the osteogenic differentiation of PDLSCs, we suppressed Smad5 using siRNA and the knockdown efficiency was verified by real-time RT-PCR and western blot. Targets of Si-Smad5-3 and Si-Smad5-4 obviously inhibited the expression of Smad5 (Fig. 5A,B). We choose the target of Si-Smad5-3 as subsequent formal experiment. Assessment of ALP activity and Alizarin Red S staining showed that ALP activity level was much lower (Fig. 5C,D) and matrix mineralization was decreased in the hPDLSCs silencing Smad5 compared to the Si-control and the untransfected cells (Fig. 5C,E). On the other hand, we observed that silencing Smad5 inhibited ALP, BSP, Runx2 and OSX gene expression after 7 days of osteogenic induction (Fig. 5F–I). Furthermore, Runx2 expression was down-regulated on a protein level in the Smad5-knockdown cells compared to the Si-control and untransfected cells (Fig. 5J). Moreover, Smad5 is an upstream regulator of Runx2, further to elevated levels of osteobalstic genes, including Runx2. We show that inhibition of miR-21 promotes osteoblastic differentiation can be attributed to stimulating Smad5-Runx2 signaling way (Fig. 5K).

## Discussion

An increasing body of literatures has indicated that miRNAs could serve as key regulators of MSC osteogenesis. For instance, it has been suggested that miR-320a could prevent hMSCs from differentiating into osteoblasts by targeting homeobox a10 (HOXA10)^22^. In another study, miR-196a was shown to enhance the osteogenic differentiation and decrease the proliferation of hASCs through targeting HOXC8^23^. Recently, miR-21 was reported to be able to stimulate the osteogenic differentiation of human umbilical cord MSCs via the PI3K/β-catenin pathway^24^. Despite these findings, the effect of miR-21 on the osteogenic differentiation of hPDLSCs, a new population of MSCs derived from dental and craniofacial tissues^1^, has not been elucidated. Our current study found a decreased expression level of miR-21 in osteoblastically differentiated hPDLSCs, implying its role as an inhibitor of MSC osteogenesis. In agreement with this finding, several osteogenic genes, including ALP, Runx2, BSP and OSX, were down-regulated in hPDLSCs overexpressing miR-21. Conversely, the opposite results were obtained when the expression of miR-21 was suppressed. Our study also identified and experimentally verified Smad5 as a potential downstream target of miR-21, suggesting the former could be mechanistically implicated in the regulation of osteogenic differentiation in PDLSCs. It is worth noting that our experimental results were not consistent with those mentioned in Meng’s study, which suggested miR-21 as a stimulator of osteogenic differentiation in human umbilical cord MSCs^24^. We have previously demonstrated that external mechanical stretch could up-regulate miR-21 in osteoblastically differentiated PDLSCs in a force-dependent manner^18^. It is very plausible that the exact regulatory function of miR-21 could vary according to the type of stem cell used and the physiological conditions applied.

To study the molecular mechanism by which miR-21 regulates hPDLSC osteogenesis, we searched for potential target genes that have been identified as critical transcription factors in osteogenic differentiation. Our bioinformatics analysis led to the identification of Smad5, which has been established to mediate the bone morphogenetic protein (BMP) signaling pathway^25–27^. A number of literature reports have associated Smad5 with the regulation of osteogenesis. For example, Li *et al*. showed that Smad5 was mechanistically implicated in miR-135′s inhibition of multiple components that were deemed necessary for the osteogenesis of C2C12 mesenchymal cells^28^. In another study, the suppression of the osteogenic differentiation of hBMSCs by miR-222-3p was revealed to involve the modulation of the Smad5-Runx2 signaling axis. In addition, as the upstream regulator, Smad5 has been shown to promote the expression of Runx2 and several other osteogenic marker genes such as ALP, OSX and OCN^29^. Our current study showed that overexpression of miR-21 down-regulated the protein level of Smad5 seven days after the hPDLSCs were induced to differentiate. Conversely, inhibition of miR-21 increased Smad5 expression and inhibited the tendency of hPDLSCs toward osteogenic differentiation. Most important of all, luciferase reporter assay suggested that miR-21 mediated osteogenic differentiation effects by directly targeting Smad5. These data, combined with our animal experiments, strongly implied Smad5 to be a downstream target of endogenous miR-21 and serve as a key regulator for osteogenic differentiation in hPDLSCs.

Next, we analyzed the effects of Smad5 modulation on PDLSCs differentiation. miR-21 level increased after knockdown of Smad5. While the expression of osteogenic marker genes such as ALP, BSP, Runx2 and OSX decreased. Thus, Smad5 suppression mimic the effects of miR-21 overexpression. As mentioned before, Runx2 was found to be up-regulated by and acted as a downstream effector of Smad5^29^. Accordingly, the protein level of Runx2 decreased in Smad5 inhibitor group, strongly that Runx2 was regulated by Smad5. Therefore, we speculated that the Smad5-Runx2 signaling pathway could constitute a critical component underlying the miR-21 regulatory machinery on the osteoblastic differentiation of hPDLSCs, as schematically illustrated in Figure 5K. However, other unidentified miR-21 targets could also contribute to the phenotypic alterations observed upon the inhibition or overexpression of miR-21. In fact, we have noticed several additional genes related to osteogenic differentiation, including sex determining region Y-box 2, activated protein C, cyclin-dependent kinase 6, as well as microphthalmia-associated transcription factor, that were predicted to be the downstream targets of miR-21. The involvement of these potential osteogenic targets should be verified in future studies.

There is also mounting evidence that other miRNAs might play important regulatory roles in the osteogenic differentiation of different types of stem cells. For example, inhibition of miR-26a activity promoted osteogenic differentiation of human adipose tissue-derived stem cells (hADSCs) via Smad1^30^. MiR-135b was found to inhibit the osteogenic differentiation of human MSCs by suppressing the expression of Smad5^21^. Furthermore, miR-26b and miR-29b were up-regulated during in osteoblastically differentiated unrestricted somatic stem cells (USSC) and inhibited their osteogenesis by modulating the levels of Smad1 and Smad6, respectively^31^. These findings are consistent with the widely accepted theory that miRNAs, their target mRNAs and other non-coding RNAs form an extremely intricate regulatory network, in which one miRNA can target multiple mRNAs and different miRNAs can modulate the same mRNA.

In summary, we have demonstrated that miR-21 could down-regulate the osteogenic differentiation of hPDLSCs by targeting Smad5. Importantly, functional inhibition of miR-21 could accelerate osteogenic differentiation of hPDLSCs and lead to increased bone formation *in vivo*. Our results could provide mechanistic insights into the molecular process of stem cell differentiation. In addition, we are convinced that the targeting of miR-21 could serve as a promising therapeutic tool for stimulating bone formation and mitigating pathological conditions associated with bone loss.

## Materials and Methods

### Ethics statement

All protocols for handling dental tissues were performed in accordance with relevant guidelines and regulations. This study was approved by the Research Ethics Committee of Shandong University (NO.MECSDUMS2012087). Informed consent was obtained from the donors and their parents. All animal experiments were conducted under the guide of the Care and Use of Laboratory Animals of the Chinese Science and Technology Ministry. This study was approved by the Committee on the Ethics of Animal Experiments of Shandong University (NO.ECAESDUSM2012075). All procedures were performed under pentobarbital sodium anesthesia.

### Cell Culture

The study included a total of 24 healthy human first premolars extracted for orthodontic reasons from 12 donors aged 10–14 years at the Department of Oral Maxillofacial Surgery, School of Stomatology, Shandong University. The teeth were first disinfected with 75% ethanol and then washed with phosphate buffered saline (PBS). PDLSCs were isolated, cultured, and identified, as previously described^1,17^. Briefly, hPDLSCs were gently separated from the middle third of the root surface of the first premolars and then digested in a solution of 3 mg/mL collagenase type I (Sigma-Aldrich Corp., St. Louis, MO, USA) and 4 mg/mL dispase II (Sigma-Aldrich Corp., St. Louis, MO, USA) for 1 h at 37 °C. Samples from different individuals were pooled and single-cell suspensions were obtained by passing the cells through a 70 µm strainer (Falcon, BD Labware, Franklin Lakes, NJ, USA). Cells were cultured in alpha-modification of Eagle’s medium (Gibco; Invitrogen Corp., Carlsbad, CA, USA) supplemented with 15% fetal calf serum (Gibco; Invitrogen Corp., Carlsbad, CA, USA), 2 mmol/L glutamine, 100 U/mL penicillin and 100 μg/mL streptomycin (Invitrogen Corp., Carlsbad, CA, USA) in a humidified incubator under 5% CO_2_ at 37 °C. The third or fourth passage of PDLSCs was used in the following experiments.

### Small interfering RNA (siRNA)

The control siRNA (Si-control) and siRNA duplexes specific for Smad5 (Si-Smad5) were purchased from Oligobio (Oligobio CO., LTD. Beijing, China). Four interference targets are designed (Si-Smad5-1, Si-Smad5-2, Si-Smad5-3, Si-Smad5-4). SiRNAs were transfected into hPDLSCs using ribo*FECT* ^TM^ CP according to the manufacturer’s instructions (GUANGZHOU RIBIO CO., LTD. Guangzhou, China). Total RNA and proteins were extracted from the siRNA-treated cells after 72 h of transfection. RT-PCR and Westernblot were used to verify the interference efficiency.

### Plasmid Construction and Viral Infection

The following lentiviral plasmid constructs were designed and were constructed by Shanghai Genechem Co. LTD. (Shanghai, China): pGC-LV-pre-miR-21-GFP (LV-miR21) for the overexpression of hsa-miR-21-5p, empty vector GC-LV-miR-NC-GFP (LV-NC) used as a control for LV-miR-21-GFP, pGC-LV-anti-miR-21-GFP (LV-anti-miR21) for the inhibition of hsa-miR-21-5p, and pGC-LV-anti-miR-NC-GFP (LV-anti-NC) used as a control for anti-miR-21-GFP. PDLSCs were seeded at a density of 2.5 × 10^5^ cells per cm^2^ in 6-well plates. When the cells reached 80% confluence, lentiviral transduction was conducted at a multiplicity of infection of 20 according to the manufacturer’s instructions. The forward primers used for the transduction were as follows: 5′-CGGCCGCGACTCTAGTTATCAAATCCTGCCTGACTG-3′for hsa-miR-21-5p, and 5′-CCGGTTCAACATCAGTCTGATAAGCTATTTTTG-3′ for the inhibition of hsa-miR-21-5p.

### Induction of osteogenic differentiation

HPDLSCs were grown in mineralization-inducing medium containing 100 μM ascorbic acid, 2 mM β-glycerophosphate and 10 nM dexamethasone. For ALP staining, after 7 days of induction, hPDLSCs were fixed with 4% paraformaldehyde and stained with a solution of 0.25% naphthol AS-BI phosphate and 0.75% fast red violet (supplied in the ALP Activity Kit). ALP activity was measured using the ALP Activity Kit according to the manufacturer′s protocol (Sigma-Aldrich Corp., St. Louis, MO, USA) and was normalized to the total protein concentration in the sample.

To detect mineralization, cells were induced for 3 weeks, fixed with70% ethanol, and stained with 2% Alizarin red S (Sigma-Aldrich Corp., St. Louis, MO, USA). To quantify the concentration of calcium, Alizarin Red S was de-stained with 10% cetylpyridinium chloride in 10 mM sodium phosphate for 30 min at room temperature. The absorbance at 562 nm was measured on a multiplate reader and the calcium level was calculated from the obtained value using a standard curve constructed based on calcium serial dilutions prepared in the same solution. The final calcium level was normalized to the total protein concentration in the sample, measured in duplicate^32^.

### Luciferase assay

Luciferase assays were completed by RIBO Company (GUANGZHOU RIBIO CO., LTD. Guangzhou, China). 1.5 × 10^4^ 293 T Cells were plated in 96-well plates at 24 h before transfection. After transfection with pGC-LV-pre-miR-21-GFP or pGC-LV-GFP, the 3′UTR vector or mutant vector of target gene were co-transfected into cells using Lipofectamine TM 2000 reagent (GUANGZHOU RIBIO CO., LTD. Guangzhou, China). We designed two mutant vectors (Smad5-MUT-1, Smad5-MUT-2). After transfection for 48 h, luciferase substrate was added and shaken for 10 min to determine the fluorescence value. Then added 30 μL Stop reagent, shake for 10 min, and the luciferase activities were measured with with the Dual-Luciferase Reporter system (Promega). Each experiment was performed in triplicate.

### RNA extraction and real-time PCR

To measure the mRNA levels of miR-21 and other osteogenic genes, total RNA was isolated from PDLSCs using RNAiso TM Plus (TAKARA Bio Inc., Otsu, Shiga, Japan). After DNase treatment, 1 µg of the total RNA was reverse-transcribed using has-miR21 qPCR Primer Mix and PrimeScript^®^ RT Reagent Kit With gDNA Eraser (TAKARA Bio Inc., Otsu, Shiga, Japan). Relative transcript levels were measured by qRT-PCR in a 20 µL reaction mixture consisting of 10 μL SYBR^®^Premix Ex Taq^TM^ (Tli RNaseH Plus, TAKARA Bio Inc., Otsu, Shiga, Japan), 0.4 μL 10 μM forward primer (to a final concentration of 200 nM), 0.4 μL 10 μM reverse primer (to a final concentration of 200 nM) and 100 ng template DNA in DEPC-treated water. PCR was conducted in a Roche Light Cycler^®^ 480 Sequence Detection System (Roche Diagnostics GmbH, Mannheim, Baden-Württemberg, Germany). U6 snRNA or glyceraldehyde 3-phosphate dehydrogenase was used as an internal control for result normalization. Primers for U6 (D356-03) and miR-21 (DHM0189) were purchased from TAKARA Bio Inc. Primer pairs used for qPCR amplifications are listed in Table 2. The PCR program was as follows: 95 °C for 30 s, then 40 cycles of 95 °C for 5 s, 60 °C for 20 s and 65 °C for 15 s. The specificity of the reaction is evaluated by determining the Tms of the amplification products immediately after the last amplification cycle. The value 2^−∆∆CT^ was used for comparative quantitation. All PCRs were performed in triplicates.

**Table 2:** The primers used for PCR.

Gene name	Primer (5′ to 3′)	Primer sequence
miR-21	Forward	GCGGCGTAGCTTATCAGACT
	Reverse	AGTGCAGGGTCCGAGGTATT
U6	Forward	CTCGCTTCGGCAGCACA
	Reverse	AACGCTTCACGAATTTGCGT
BSP	Forward	ACGATTTCCAGTTCAGGGCA
	Reverse	TCCTCTCCATAGCCCAGTGT
Runx2	Forward	CGAATAACAGCACGCTATTAA
	Reverse	GTCGCCAAACAGATTCATCCA
OSX	Forward	ACCTACCCATCTGACTTTGCTC
	Reverse	CCACTATTTCCCACTGCCTTG
ALP	Forward	TAGTGAAGAGACCCAGGCGCT
	Reverse	ATAGGCCTCCTGAAAGCCGA
Smad5	Forward	CGGTAGCCAACTGACTTTGAGT
	Reverse	ACCTTGTTTCCAGCCCAACA
GAPDH	Forward	TCATGGGTGTGAACCATGAGAA
	Reverse	GGCATGGACTGTGGTCATGAG

### Western blot analysis

Two sets of Western blot experiments were conducted as follows. On the one hand, untransfected hPDLSCs were grown as described above for 0, 1, 3, 5, 7 d, respectively and then lysed using RIPA lysis buffer (Beyotime). On the other hand, different groups of transfected hPDLSCs (see Plasmid construction and viral infection for more information on the lentiviral constructs used) were grown for 7 d prior to lysis. In both cases, the total protein concentrations in the lysate samples were measured by the Bio-Rad Protein Assay Kit (Bio-Rad Laboratories, Hercules, CA, USA). The proteins in the lysate samples were separated by electrophoresis on 10% SDS-PAGE gels under a constant voltage of 80 V for 2 h and then transferred onto polyvinyldifluoride (PVDF) membranes (Amersham Biosciences, Uppsala, Uppland, Sweden). The membranes were blocked for 45 to 90 min at room temperature using 5% (v/v) skimmed milk in Tris-buffered saline solution with Tween-20 (TBS-T), following by incubation with anti-Smad5 (1:500) (Sigma-Aldrich Corp., St. Louis, MO, USA) and anti-Runx2 (1:500) (Abcam, Cambridge, UK) as the primary antibody at 4 °C overnight. After three 5-min washes in TBS-T, the membranes were incubated at room temperature for 2 h with 1:5000 peroxidase-conjugated anti-rabbit IgG (Jackson ImmunoReserach Inc., West Grove, Pennsylvania, USA). Immunoreactive bands were detected using the Enhanced Chemiluminescence Plus Detection Kit (GE Healthcare, Little Chalfont, Buckinghamshire, UK) according to the manufacturer’s instructions.

### Transplantation in Nude Mice

Approximately 4.0 × 10^6^ cells were resuspended in 500 μL medium and mixed with 40 mg of HA/TCP ceramic particles obtained from the Engineering Research Center for Biomaterials, Sichuan University, China. The resultant mixtures were then implanted subcutaneously into the dorsal surface of 10-week-old immunocompromised beige mice (nu/nu nude mice) as previously described^33,34^. These procedures were performed in accordance the approved animal protocol. Eight weeks after the implantation procedures, the implants were removed and fixed with 10% formalin, decalcified in 10% EDTA (pH 8.0), and then embedded in paraffin. Samples were cut into 4μm sections and stained with H&E stain for immunohistochemical analyses. The protein level of Smad5 was determined by immunostaining using the ALK Complex Kit (Zhongshan Golden Bridge Biological Technology Co. Ltd., Beijing, China) following the manufacturer’s instructions. Briefly, the sections were deparaffinized and then permeabilized in PBS. After rinsing, the sections were incubated with 1:500 rabbit anti-Smad5 (Sigma-Aldrich Corp., St. Louis, MO, USA) in phosphate buffered saline at 37 °C overnight. Next, the sections were incubated with 1:5000 biotinylated goat anti-rabbit IgG (Jackson ImmunoReserach Inc., West Grove, Pennsylvania, USA) in phosphate buffered saline for 2 h at room temperature. Finally, the specimens were visualized using 3–3′-diaminobenzidine (DAB, Wako, Osaka, Japan) and counterstained with hematoxylin. All images were captured using a CCD camera (CS600, Olympus, Tokyo, Japan).

### Statistical analysis

All statistical calculations were performed using SPSS10.0 (SPSS Inc., Chicago, IL, USA). All data were normally distributed and presented as the mean ± standard deviation of three to five independent experiments. Differences between the results obtained from various experimental groups were analyzed by the student’s t test or one-way analysis of variance (ANOVA). A *p* value less than 0.05 was considered statistically significant and was adjusted by the Bonferroni method to allow for multiple comparisons.

## Electronic supplementary material

Supplementary Information

## References

[1] Seo BM (2004). *Investig*ation of multipotent postnatal stem cells from human periodontal ligament. Lancet.

[2] Liu Y (2008). Periodontal ligament stem cell-mediated treatment for periodontitis in miniature swine. Stem Cells.

[3] Liu O (2013). Periodontal ligament stem cells regulate B lymphocyte function via programmed cell death protein 1. Stem Cells.

[4] Ding G (2010). Allogeneic periodontal ligament stem cell therapy for periodontitis in swine. Stem Cells.

[5] Wei F (2012). Vitamin C treatment promotes mesenchymal stem cell sheet formation and tissue regeneration by elevating telomerase activity. J Cell Physiol.

[6] Wei F (2013). Functional tooth restoration by allogeneic mesenchymal stem cell-based bio-root regeneration in swine. Stem Cells Dev.

[7] Takeuchi Y (1997). Differentiation and transforming growth factor-beta receptordown-regulationby collagen-alpha2beta1 integrin interactionismediatedby focaladhesionkinase and its downstream signals inmurine osteoblastic cells. J Biol Chem.

[8] Salasznyk RM, Klees RF, Williams WA, Boskey A, Plopper GE (2007). Focal adhesionkinase signaling pathways regulate the osteogenic differentiation of human mesenchymal stem cells. Exp Cell Res.

[9] Yi R, Poy MN, Stoffel M, Fuchs E (2008). A skin microRNA promotes differentiation by repressing ‘stemness’. Nature.

[10] Lian JB (2012). MicroRNA control of bone formation and homeostasis. Nat Rev Endocrinol.

[11] Jing D (2015). The role of microRNAs in bone remodeling. Int J Oral Sci.

[12] Volinia S (2006). A microRNA expression signature of human solid tumors defines cancer gene targets. Proc Natl Acad Sci USA.

[13] Si ML (2007). miR-21-mediated tumor growth. Oncogene.

[14] Asangani IA (2008). MicroRNA-21 (miR-21) post-transcriptionally downregulates tumor suppressor Pdcd4 and stimulates invasion, intravasation and metastasis in colorectal cancer. Oncogene.

[15] Meng F (2007). MicroRNA-21 regulates expression of the PTEN tumor suppressor gene in human hepatocellular cancer. Gastroenterology.

[16] Zhu S (2008). MicroRNA-21 targets tumor suppressor genes in invasion and metastasis. Cell Res.

[17] Fujita S (2008). miR-21 Gene expression triggered by AP-1 is sustained through a double-negative feedback mechanism. J Mol Biol.

[18] Wei FL (2014). Mechanical Force-Induced Specific MicroRNA Expression in Human Periodontal Ligament Stem Cells. Cells Tissues Organs.

[19] Choi MH, Noh WC, Park JW, Lee JM, Suh JY (2011). Gene expression pattern during osteogenic differentiation of human periodontal ligament cells *in vitro*. J Periodontal Implant Sci.

[20] Choi HD, Noh WC, Park JW, Lee JM, Suh JY (2011). Analysis of gene expression during mineralization of cultured human periodontal ligament cells. J Periodontal Implant Sci.

[21] Xu S (2013). Upregulation of miR-135b is involved in the impaired osteogenic differentiation of mesenchymal stem cells derived from multiple myeloma patients. PLoS One.

[22] Huang J (2016). MicroRNA-320a Regulates the Osteogenic Differentiation of Human Bone Marrow-Derived Mesenchymal Stem Cells by Targeting HOXA10. Cell Physiol Biochem.

[23] Kim YJ, Bae SW, Yu SS, Bae YC, Jung J (2009). S. miR-196a regulates proliferation and osteogenic differentiation in mesenchymal stem cells derived from human adipose tissue. J Bone Miner Res.

[24] Meng YB (2015). microRNA-21 promotes osteogenic differentiation of mesenchymal stem cells by the PI3K/β-catenin pathway. J Orthop Res.

[25] Fujii M (1999). Roles of bone morphogenetic protein type I receptors and Smad proteins in osteoblast and chondroblast differentiation. Mol Biol Cell.

[26] Lee KS (2000). Runx2 is a common target of transforming growth factor beta1 and bone morphogenetic protein 2, and cooperation between Runx2 and Smad5 induces osteoblast-specific gene expression in the pluripotent mesenchymal precursor cell line C2C12. Mol Cell Biol.

[27] Nishimura R, Hata K, Harris SE, Ikeda F, Yoneda T (2002). Core-binding factor alpha 1 (Cbfa1) induces osteoblastic differentiation of C2C12 cells without interactions with Smad1 and Smad5. Bone.

[28] Li Z (2008). A microRNA signature for a BMP2-induced osteoblast lineagecommitment program. Proc NatlAcad Sci USA.

[29] Yan J (2016). Inhibition of miR-222-3p activity promoted osteogenic differentiation of hBMSCs by regulating Smad5-RUNX2 signal axis. Biochem Biophys Res Commun.

[30] Luzi E (2008). Osteogenic differentiation of human adipose tissue-derived stem cells is modulated by the miR-26a targeting of the SMAD1 transcription factor. J Bone Miner Res.

[31] Trompeter HI (2013). MicroRNAs miR-26a, miR-26b, and miR-29b accelerate osteogenic differentiation of unrestricted somatic stem cells from human cord blood. BMC Genomics.

[32] Fan, Z. *et al*. BCOR regulates mesenchymal stem cell function by epigenetic mechanisms.*Nat Cell Biol* **11**, 1002–1009, 10.1038/ncb1913(2009). 10.1038/ncb1913 PMC2752141 19578371

[33] Yamaza T (2009). Mesenchymal stem cell-mediated ectopic hematopoiesis alleviates aging-related phenotype in immunocompromised mice. Blood.

[34] Fujita A (2010). Hematopoiesis in regenerated bone marrow within hydroxyapatite scaffold. Pediatr Res.

