# Supplementary material for: MicroRNA-21 regulates Osteogenic Differentiation of Periodontal Ligament Stem Cells by targeting Smad5
Source: Sci Rep. 2017 Nov 30;7:16608. doi: 10.1038/s41598-017-16720-8 (PMC5709498; doi:10.1038/s41598-017-16720-8)
Supplement: Supplementary file 1 — Supplementary Information [file 41598_2017_16720_MOESM1_ESM.docx]

**MicroRNA-21 regulates Osteogenic Differentiation of Periodontal Ligament Stem Cells by targeting Smad5**

Fulan Wei^1,2*^, Shuangyan Yang^1,2^, Qingyuan Guo^2^, Xin Zhang^1^, Dapeng Ren^2^, Tao Lv^1,2^ & Xin Xu^2,3*^

1. Department of Orthodontics, School of Stomatology, Shandong University, Jinan, People’s Republic of China.

2. Shandong Provincial Key Laboratory of oral tissue regeneration, School of Stomatology, Shandong University, Jinan, People’s Republic of China.

3.Department of Implantology, School of Stomatology, Shandong University, Jinan, People’s Republic of China.

*Correspondence: F. W. (weifl@sdu.edu.cn) or X. X (email: [xinxu@sdu.edu.cn](mailto:xinxu@sdu.edu.cn))

Supplementary Figures


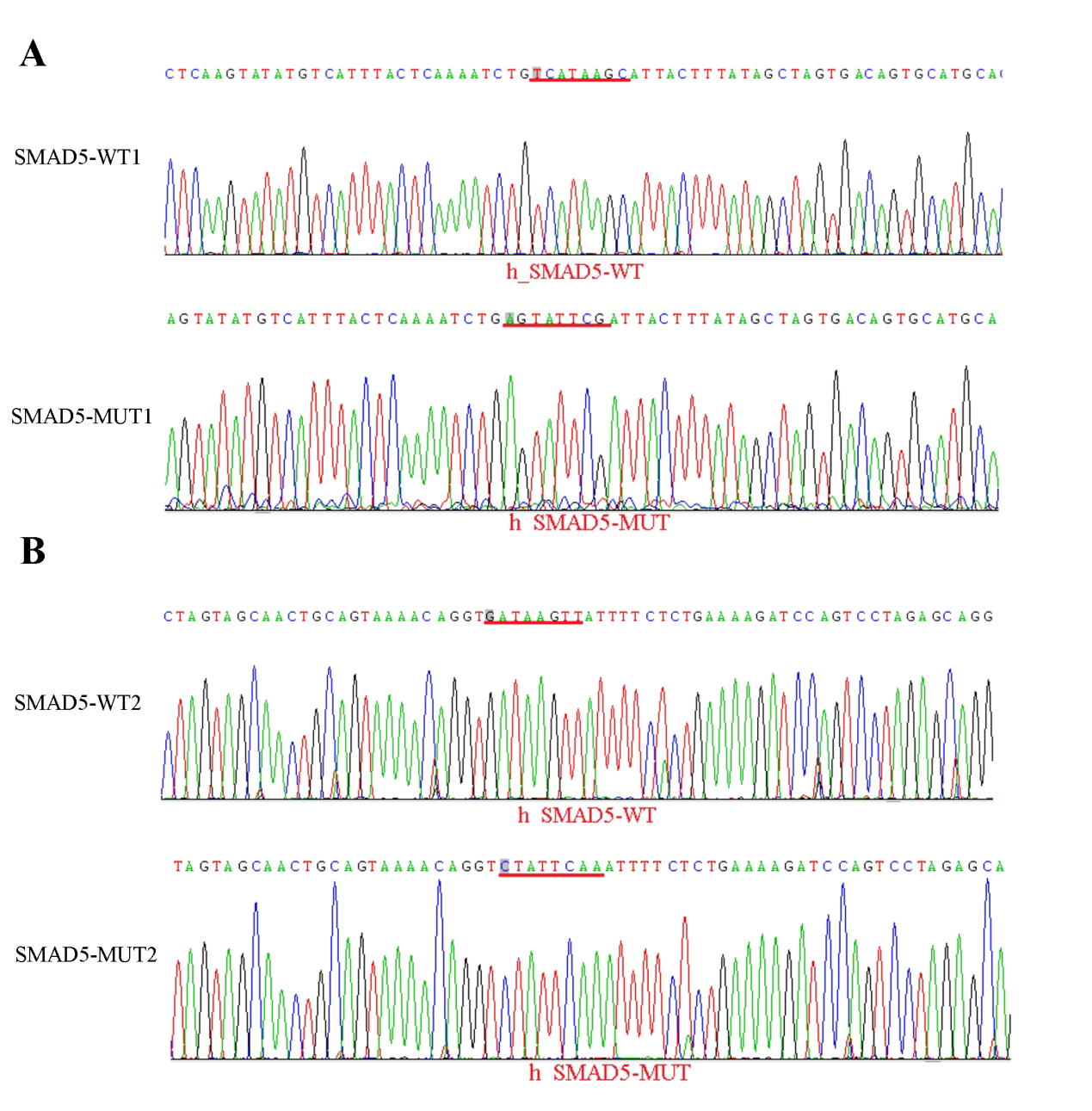


**Figure S1. The sequencing results of the Smad5-mutant vectors (Smad5-MUT-1, Smad5-MUT-2). (A) T**he sequencing results of the Smad5-MUT-1: TCATAAGC (730-737) mutated to AGTATTCG. (B) The sequencing results of the Smad5-MUT-2: GATAAGTT (4459-4466) mutated to CTATTCAA.AGTATTCG.


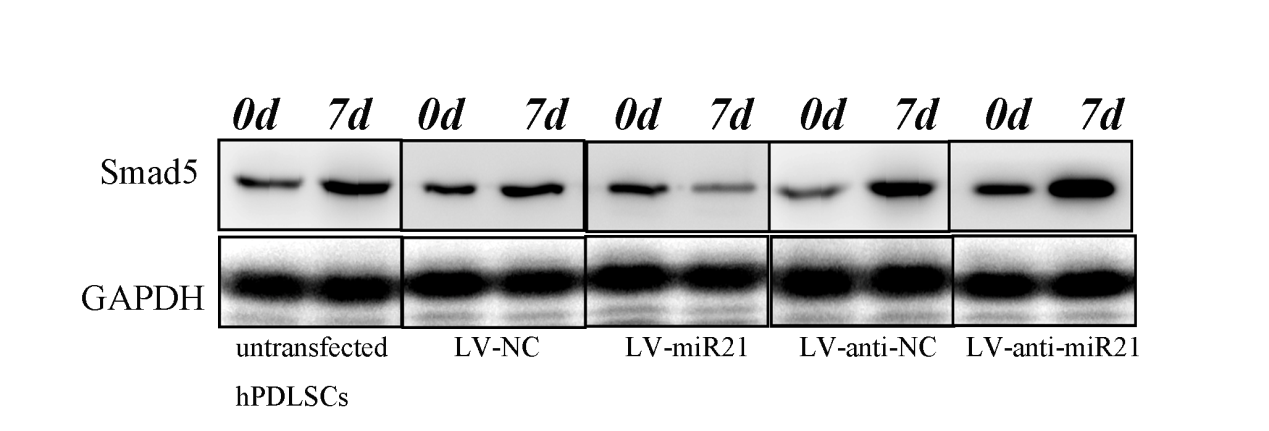


**Figure S2. The protein level of Smad5 after miR-21 overexpression and inhibition.** Western blot analysis indicating the expression levels of Smad5 in different experiment groups. Smad5 expression was increased in miR-21-knockdown hPDLSCs (rightmost, anti-miR21) while suppressed in those overexpressing miR-21 (middle, LV-miR21).







**Figure S3. The original scans of Figure 5B. The raw blots of Smad5 after transfection with control siRNA, siRNA-Smad5 for 72 h in PDLSCs. (Left: GAPDH; right: Smad5).**


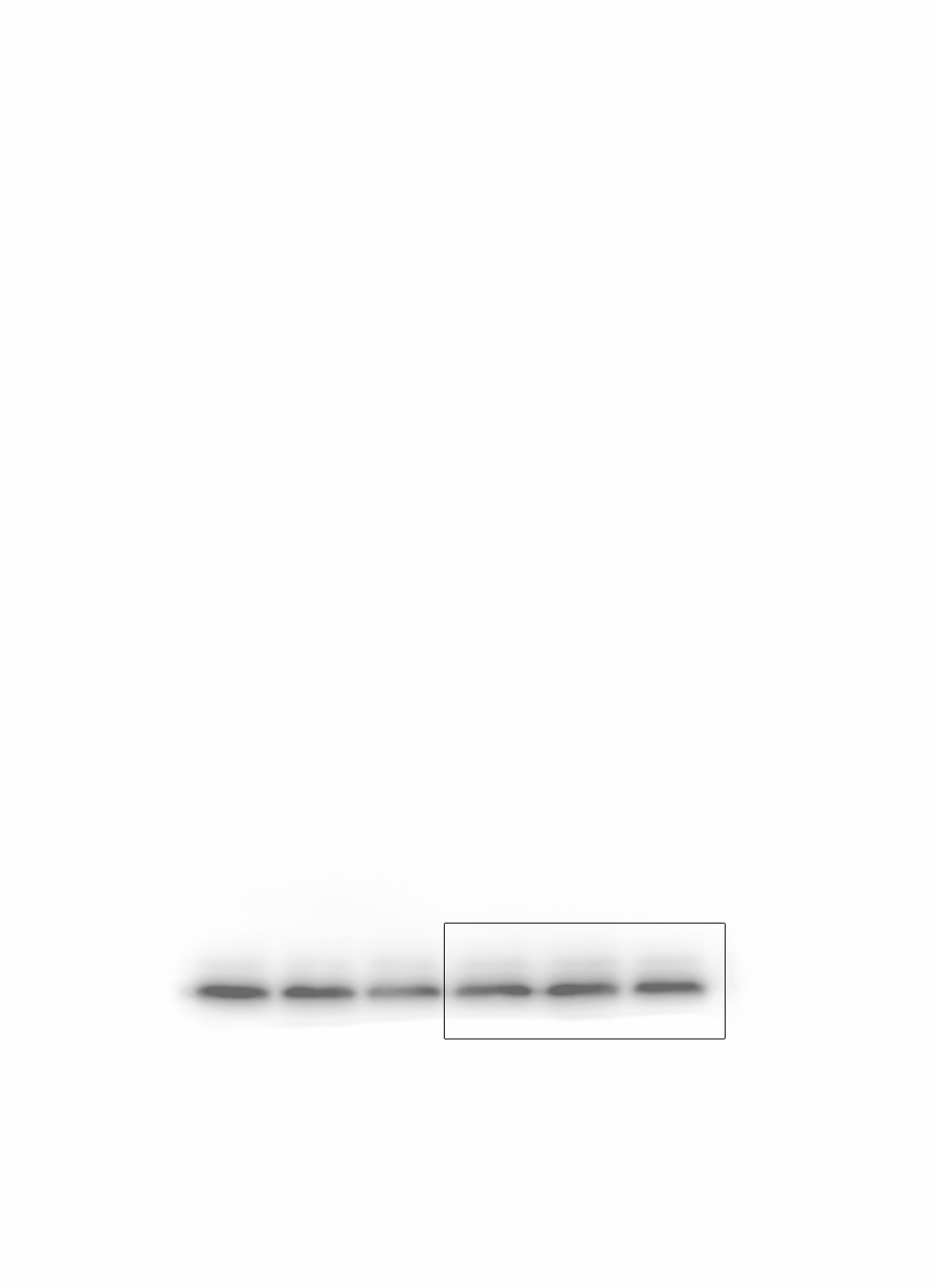

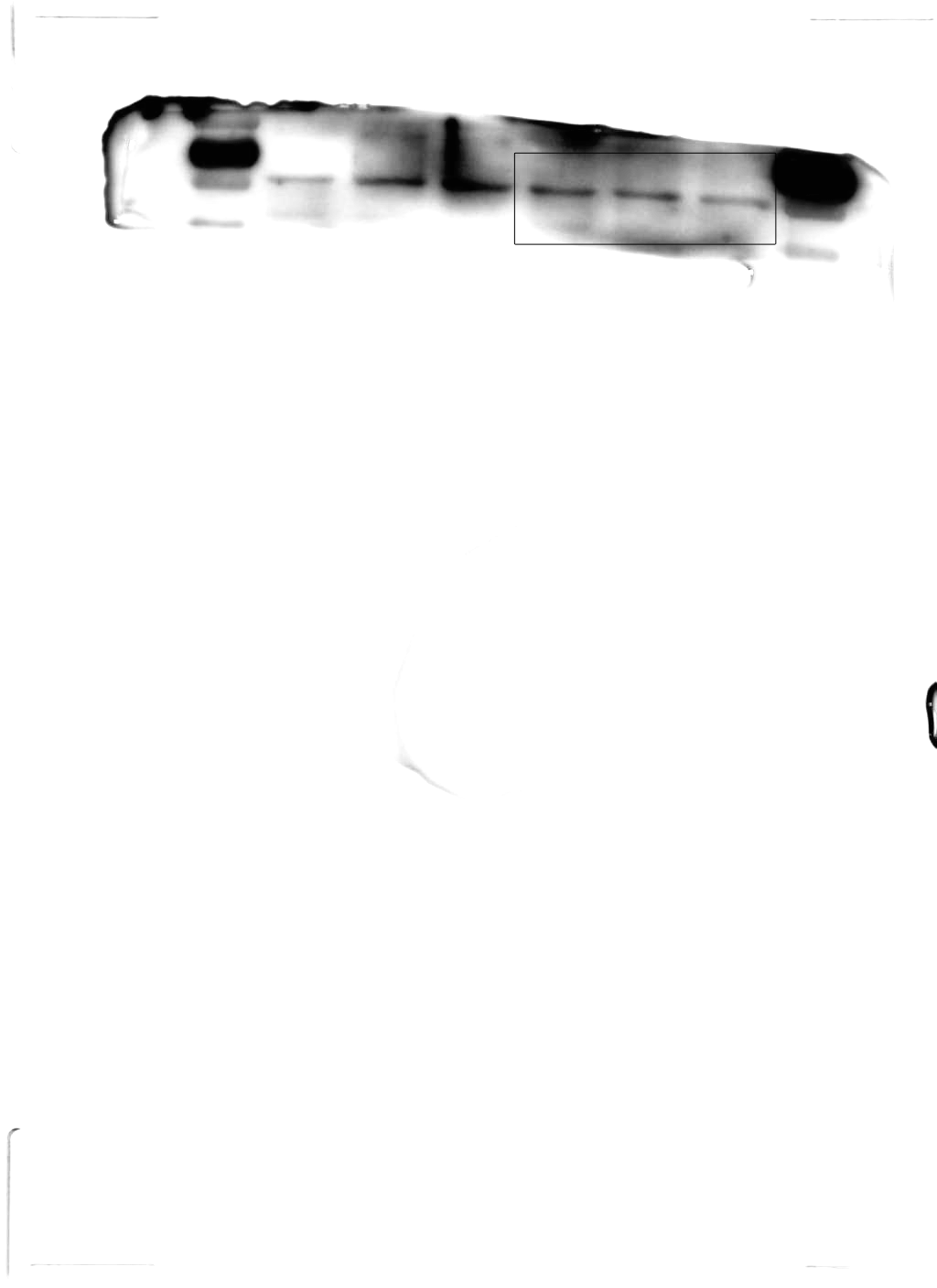


**Figure S4. The original scans of Figure 5J. (Tagged black frame). The raw blots of Runx2 after transfection with control siRNA, siRNA-Smad5 in PDLSCs. (Left: GAPDH; right: Runx2).**
